# Improvement of a Green’s Function Estimation for a Moving Source Using the Waveguide Invariant Theory

**DOI:** 10.3390/s24175782

**Published:** 2024-09-05

**Authors:** Daehwan Kim, Donghyeon Kim, Gihoon Byun, Jeasoo Kim, Heechun Song

**Affiliations:** 1 Department of Ocean Engineering, Korea Maritime and Ocean University, Busan 49112, Republic of Korea; oceankim823@gmail.com (D.K.); jskim@kmou.ac.kr (J.K.); 2 Scripps Institution of Oceanography, La Jolla, CA 92093-0238, USA; donghyeon.ual@gmail.com (D.K.); hcsong@ucsd.edu (H.S.); 3 Department of Convergence Study on the Ocean Science and Technology, Korea Maritime and Ocean University, Busan 49112, Republic of Korea

**Keywords:** Green’s function, waveguide invariant, ray-based blind deconvolution

## Abstract

Understanding the characteristics of underwater sound channels is essential for various remote sensing applications. Typically, the time-domain Green’s function or channel impulse response (CIR) is obtained using computationally intensive acoustic propagation models that rely on accurate environmental data, such as sound speed profiles and bathymetry. Ray-based blind deconvolution (RBD) offers a less computationally demanding alternative using plane-wave beamforming to estimate the Green’s function. However, the presence of noise can obscure low coherence ray arrivals, making accurate estimation challenging. This paper introduces a method using the waveguide invariant to improve the signal-to-noise ratio (SNR) of broadband Green’s functions for a moving source without prior knowledge of range. By utilizing RBD and the frequency shifts from the striation slope, we coherently combine individual Green’s functions at adjacent ranges, significantly improving the SNR. In this study, we demonstrated the proposed method via simulation and broadband noise data (200–900 Hz) collected from a moving ship in 100 m deep shallow water.

## 1. Introduction

Characterizing the wave propagation in an ocean waveguide is very important in various acoustic applications such as underwater communication [1], geoacoustic inversion [2,3], matched field processing [4,5,6,7], etc. The Green’s function also referred to as the channel impulse response (CIR), describes the broadband sound field between the source and receiver. Because of the complexity of the ocean environment, accurately identifying the Green’s function is a challenging problem. The Green’s function can be obtained using various acoustic propagation models [8] with the environmental parameters (e.g., sound speed profile (SSP), geoacoustic parameters, and bathymetry). However, obtaining the Green’s function using propagation models normally requires high computational costs due to broadband calculations and could result in mismatches due to inaccurate environmental information [9,10].

Estimation of the Green’s function from the measured data minimize the environmental uncertainty. Specifically acoustic fields generated from a distant source and received by a vertical array contains all the information of the channel. Ray-based blind deconvolution (RBD) can be a useful method to estimate the Green’s function with low computational complexity [11,12]. RBD employs a conventional plane-wave beamforming to identity a ray arrival direction to capture the phase of the unknown source signal (e.g., ship-radiated noise [12]). The captured phase component is then match filtered with the received signal along the array, providing an estimate of the broadband Green’s function. However, in practice, ray arrivals that have low coherence with the source phase component are masked by noise; as a result, it can be challenging to accurately estimate the Green’s function. For a stationary source, averaging multiple snapshots can mitigate this issue. However, for a moving source, the Green’s function changes over time, which limits the use of multiple snapshots.

The waveguide invariant theory introduced by Chuprov [13] showed the interference structure of broadband signals in an ocean waveguide, manifesting itself as intensity striations in the frequency-range plane. An interesting feature is that the slope of this intensity striation can be approximated as a single scalar value under certain conditions (e.g., β=1 in shallow waters) regardless of the details of ocean environmental parameters [14,15]. Recently, Song and Byun [16] derived an analytic relationship between two adjacent Green’s functions to extrapolate a Green’s function at a representative range to adjacent ranges.

The goal of this paper is to demonstrate the improvement in the SNR of the estimated Green’s function for a moving source without prior knowledge of range, SSP, and other ocean parameters. Individual Green’s functions estimated at adjacent ranges are brought back to a single point through extrapolation. Subsequently, the individual Green’s functions are coherently combined to improve the SNR of Green’s functions. With the frequency shifts obtained from the striation slope for a moving source and Green’s functions estimated via RBD, the proposed method does not require range information of individual Green’s functions to be coherently combined. We demonstrate the improvement of Green’s function via numerical simulation and experimental data.

The remainder of this paper is organized as follows: in Section 2, we review the analytic relationship between adjacent Green’s functions using the waveguide invariant and RBD [16]. Section 3 describes a recent shallow-water environment experiment (SAVEX15) [17], where the research vessel (R/V) *Onnuri* was towing two sources along a ship track in 100 m deep shallow water. Section 4 analyzes Green’s functions from a ship of opportunity radiating broadband (200–900 Hz) noise using RBD [11]. The approach for improving a Green’s function is demonstrated at a representative point of observation time 200 s in the ship track. Furthermore, the improvement is extended to the entire ship track, which is followed by a conclusion in Section 5.

## 2. Method

In this section, we describe the methods of Green’s function estimation and improvement. The estimation is accomplished using RBD from a moving ship of opportunity radiating broadband noise. The improvement of Green’s function is performed using the waveguide invariant theory without requiring knowledge of the source position and environmental parameters.

### 2.1. Estimation of Green’s Function

We used RBD for estimating Green’s functions from broadband noise. The theoretical formulation of ray-based blind deconvolution was described in earlier studies [11,12,18], so only a short summary is presented here. The goal of RBD is to estimate the unknown source signal and Green’s function using only the signals received along the array.

Assume a source produces a wide-band signal s(t). The signal in the frequency domain can be represented as

(1) $$S\left(\omega \right)=\left|S\left(\omega \right)\right|e^{i\phi_{s}\left(\omega \right)},$$

where S(ω) and $\phi_{s}\left(\omega \right)$ represent the amplitude and phase of the source signal for frequency ω, respectively. Then, the received signal $P_{j}\left(r_{j},r_{s},\omega \right)$ at the *j*th element in the array is expressed as

(2) $$P\left(r_{j},r_{s},\omega \right)=G\left(r_{j},r_{s},\omega \right)S\left(\omega \right),$$

where the Green’s function $G(r_{j},r_{s},\omega )$ is the frequency domain representation of the channel impulse response (CIR) between the source position $r_{s}$ and receiver position $r_{j}$. According to the ray theory of acoustic propagation, the CIR can be represented as the summation of *K* successive ray arrivals (where the number of propagating rays *K* is limited by physical properties of the ocean channel such as attenuation and bottom loss) [8]:

(3) $$G\left(r_{j},r_{s},\omega \right)=\sum_{k=1}^{K}\alpha_{k}e^{i\omega \left(T\left(\theta_{k}\right)+\tau_{j,k}\right)}$$

where $\alpha_{k}$ and $T(\theta_{k})$ represent the *k*th ray arrival amplitude and absolute travel time between the source and receiver array. For an N element receiver array (j=1,...,N), the time delay $\tau_{j,k}$ represents the differential travel time across the array for the selected *k*th ray. Assuming the array has sufficient angular resolution, the acoustic signal carried by the *k*th ray can be estimated by a time-delay beamformer. The output of the beamformer can be denoted as follows:

(4) $$B\left(\omega ;\theta_{k}\right)=\sum_{j=1}^{N}\exp\left\{-i\omega \tau_{j,k}\right\}P\left(r_{j},r_{s},\omega \right)\approx \left|B\left(\omega ;\theta_{k}\right)\right|\exp\left\{i\phi_{s}-i\omega T\left(\theta_{k}\right)\right\}$$

where $\tau_{j,k}$ is the local time delay of the *k*th arrival at the *j*th array element with respect to the array center.

First, RBD normalizes the amplitude of the recieved signals $\left(r_{j},r_{s},\omega \right)$ with respect to the average recorded energy to mitigate any influence of the unknown source spectrum |S(ω)|. Second, the phase of the beamformer output $B(\omega ,\theta_{k})$ is used to phase-rotate the normalized recorded signals to eliminate its dependency on the unknown phase of the source signal $\phi_{s}$ such that an estimated CIR $\hat{G}\left(\omega ;\theta \right)$ can be obtained for each receiver element j=1,...N with a time shift according to the selected *k*th ray as follows:

(5) $$\begin{array}{cc} {\hat{G}}_{j}\left(\omega ,\theta \right)\; & =\frac{P(r_{j},r_{s},\omega )}{\sqrt{\sum_{j=1}^{N}{\left|P\left(r_{j},r_{s},\omega \right)\right|}^{2}}}\exp\left\{-\arg(B\left(\omega ;\theta_{k}\right))\right\}\\ \; & =\frac{G(r_{j},r_{s},\omega )}{\sqrt{\sum_{j=1}^{N}{\left|G\left(r_{j},r_{s},\omega \right)\right|}^{2}}}\exp\left\{-i\omega T(\theta_{k})\right\} \end{array}$$

Using RBD, we can extract the Green’s function from the ship radiated broadband noise. By aligning the first arrivals of the Green’s functions extracted at different times, we can remove the time delays caused by the different ranges between the ship and receiver array.

### 2.2. Improvement of SNR

The goal of this paper is to improve the signal-to-noise ratio (SNR) of the Green’s function, which is essential for many underwater acoustic applications. Generally, multiple snapshots are averaged to use high SNR data for a stationary target in beamforming and matched-field processing. For a moving target, we cannot simply combine the multiple Green’s function since the characteristics of the Green’s functions are changing with range—in other words, with time. The basic idea is to adjust Green’s functions at adjacent time $g(t_{0}+\Delta t)$ to have the similar structure in the Green’s function of the reference time $g(t_{0})$ using the waveguide invariant theory.

The waveguide invariant theory describes the inference pattern of a broadband signal as a function of frequency and range. The interference structure in a slight shift in frequency shows similar maximum and minimum patterns in a shifted range for the same propagation condition. The slope patterns in the frequency-range plot can be expressed as a simple parameter β [13] (refer to Appendix A),

(6) $$\frac{\Delta \omega }{\omega }=\beta \frac{\Delta r}{r}.$$

Many studies have been conducted using waveguide invariant theory for explaining the propagation phenomena [8,19,20,21], in matched-field processing [22,23], and in time-reversal acoustics [24].

The procedure of improving the SNR of Green’s function involves three steps. The first step is to identify the points of adjacent Green’s functions in the striation structure of frequency versus time (i.e., range) assuming the source moves at a constant velocity. Once a reference point is selected, adjacent points are selected along the striation to which the reference point belongs. We select the adjacent points with equal time intervals at the frequency of the maximum peak of the striation.

The second step involves shifting the frequency of the Green’s function of the adjacent points to match the frequency of the reference point. Since the Green’s function is broadband, the frequency needs to be proportionally shifted. This can be achieved by resampling (i.e., temporal compression or dilation) the time-domain Green’s function according to the positive or negative frequency shift

(7) $$g\left(t_{0},\tau \right)\approx g\left(t_{0}+\Delta t,\alpha \tau \right),$$

where τ is the relative time which starts with the lead time 5 ms from the first arrival of the Green’s function. α is the resampling factor α=1+β(Δr/r)=1+(Δω/ω). The factor for down-sampling or up-sampling can be easily determined by the amount of frequency shift required to align the adjacent Green’s function with the reference Green’s function. The detailed derivations for Equation (7) can be found in Appendix B. A similar method is applied for extrapolating the Green’s function to the intended range [16] and improving the spatial correlation of the received signal by compensating for frequency shifts [25], considering the wave invariant β. The proposed method is also akin to the focal range shift method in time-reversal mirrors [24], which also use the range information to calculate the necessary frequency shift following Equation (6). However, unlike these methods, our approach does not require range information, making it suitable for situations with unknown sources and independent of the specific value of β.

The third step is coherently combining the resampled Green’s function to the reference Green’s function. By continuously adding *N* resampled adjacent Green’s functions to the reference Green’s function, we have a summed Green’s function:

(8) $$g_{s}\left(t_{0},\tau \right)=\sum_{n=-N/2}^{N/2}g\left(t_{0}+n\Delta t;\alpha_{n}\tau \right),$$

where $\alpha_{n}=1+\Delta \omega_{n}/\omega_{n}$ can be obtained from the striation slopes. If the adjacent Green’s functions are properly adjusted to the Green’s function of the reference time, the summation will increase the SNR compared to the original Green’s function of time $t_{0}$.

### 2.3. Numerical Simulation

**Figure 1:**
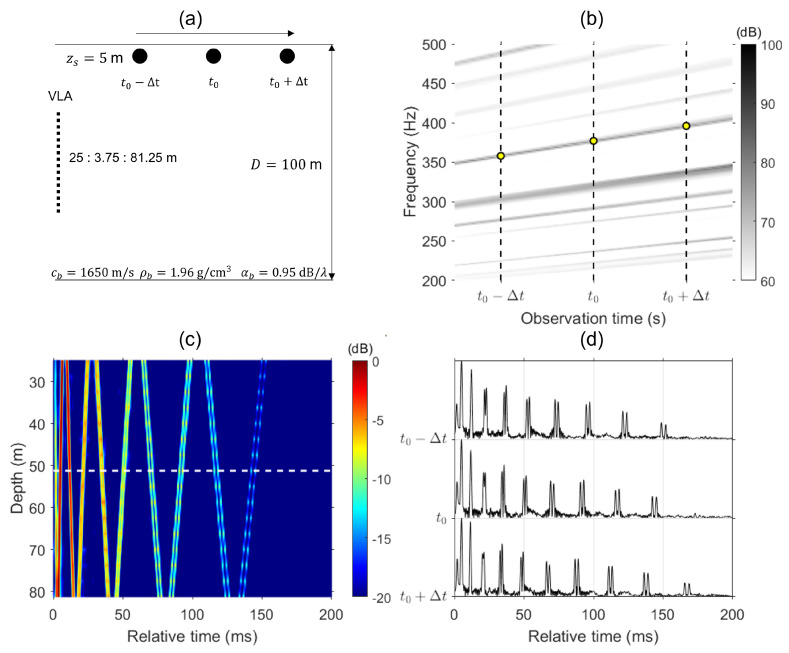
(**a**) Illustration of a Pekeris waveguide for numerical simulation. A 16-element, 56.25 m long VLA is deployed in 100 m deep shallow water with a source moving radially away from the VLA. The geoacoustic parameters are shown in the bottom. Three points with equal time separation (i.e., range separation) are presented to demonstrate the improvement of Green’s function. (**b**) Interference pattern plotted at depth d=51.25 m versus observation time and frequency. The yellow circles represent the frequency used the obtain the resampling factor α. (**c**) Green’s function estimated via RBD along the VLA at observation time $t_{0}$. (**d**) Estimated Green’s functions at depth d=51.25 m.

For numerical simulations, to demonstrate improving the SNR of Green’s functions, the normal mode program KRAKEN [26] was used, assuming a range-independent Pekeris environment with a half-space sandy bottom. The geoacoustic paramters, as indicated in Figure 1a, are density $\rho_{b}=1.97\;g/{\mathrm{cm}}^{3}$, compressional sound speed $c_{b}=1650\;m/s$, and attenuation $\alpha_{b}=0.95\;\mathrm{dB}/\lambda$. A 16-element, 56.25 m long VLA is positioned in 100 m deep shallow water assuming a source is moving radially away from the VLA at a constant speed at depth $z_{s}=5$ m. Three points with equal time separation (i.e., range separation) are selected to demonstrate the improvement of Green’s function.

As the source moves radially away from the VLA, we can observe linear striation patterns in the frequency–time plot at a depth of 51.25 m, as indicated in Figure 1b. The vertical dashed lines indicate the three points we used to demonstrate the proposed method. We obtain the frequency from the slope (350–400 Hz) at each observation time to calculate the resampling factor α=1+Δω/ω. The frequency at the reference time t0 is 377 Hz, and the frequencies at adjacent times are 358 and 396 Hz, respectively. Identifying the frequencies from the striations in the frequency–time plot is essential to the proposed method. If the noise is too severe, making the striations difficult to detect, one potential solution is to increase the FFT size (i.e., increase the FFT gain).

First, the Green’s functions are estimated via RBD. We beamform the received signal at the representative time $t_{0}$ and then steer a beam to the second angle of arrival (max beam output) and obtain the Green’s function at the reference time $g(t_{0};\tau )$ along the VLA depth, as shown in Figure 1c. Nine distinct wavefronts are observed between 0 and 200 ms with a weak 10th arrival. The white horizontal dashed line corresponds to the middle Green’s function in Figure 1d. Similarly, the Green’s functions in the neighborhood at $t_{0}-\Delta t$ and $t_{0}+\Delta t$ are estimated for combining. Figure 1d shows the estimated Green’s function in the neighborhood as well as the Green’s function at depth d=51.25 m. By eliminating the source phase from RBD with respect to the same arrival for all individual Green’s functions, all Green’s functions are aligned, allowing us to ignore the time delay of individual Green’s functions from the moving source. As the source moves away from the VLA, we can observe that the separation between arrivals narrows. Harrison [27] interpreted the behavior of striations in the frequency–range plane by exploring how the arrival times vary with range i.e., the time domain Green’s function. The fringe pattern at any given range is the Fourier transform of the impulse response. As the range changes, the time order of arrivals remains consistent, but their separation varies. As the striation slope separations increase with time (i.e., range) [see Figure 1b], the time separation of arrivals narrows.

**Figure 2:**
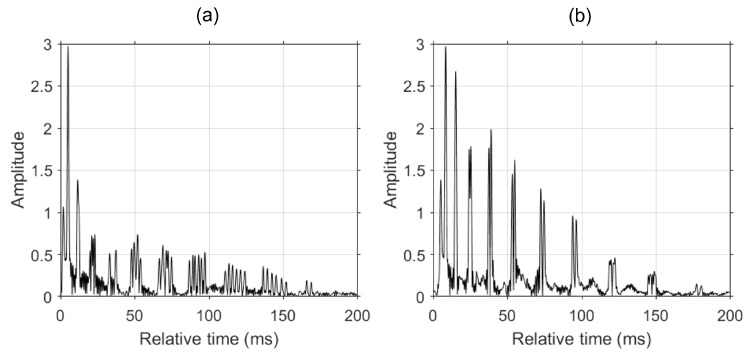
Coherently combined Green’s function (**a**) without resampling and (**b**) with resampling. The Green’s function combined with resampled adjacent Green’s functions exhibits higher amplitudes.

We now shift the frequency of the Green’s function at the adjacent points to match the frequency at the reference point with the resampling factor obtained from Figure 1b followed by coherently combining the resampled Green’s functions. When resampling the Green’s function from a closer range (i.e., $t_{0}-\Delta t$), the Green’s function requires a temporal compression. Conversely, for a further range (i.e., $t_{0}+\Delta t$), it requires instead a temporal dilation. Figure 2 compares the Green’s function coherently combined using Equation (9). Figure 2a shows the result of combining Green’s functions before resampling, while Figure 2b shows the result after. The Green’s function combined with resampled adjacent Green’s functions exhibits higher amplitudes, whereas the combined Green’s function before resampling shows poor enhancement in the later arrivals. The enhancement in the arrivals from resampled Green’s functions shows the feasibility of the proposed method.

## 3. SAVEX15 Experiment

The SAVEX15 experiment was conducted in the northeastern East China Sea using the R/V *Onnuri* in May 2015 [17]. Both fixed and towed source transmissions were carried out between two moored vertical line arrays (VLAs) over ranges of 1–10 km. The acoustic transmissions were in various frequency bands of 0.5–3.2 kHz.

**Figure 3:**
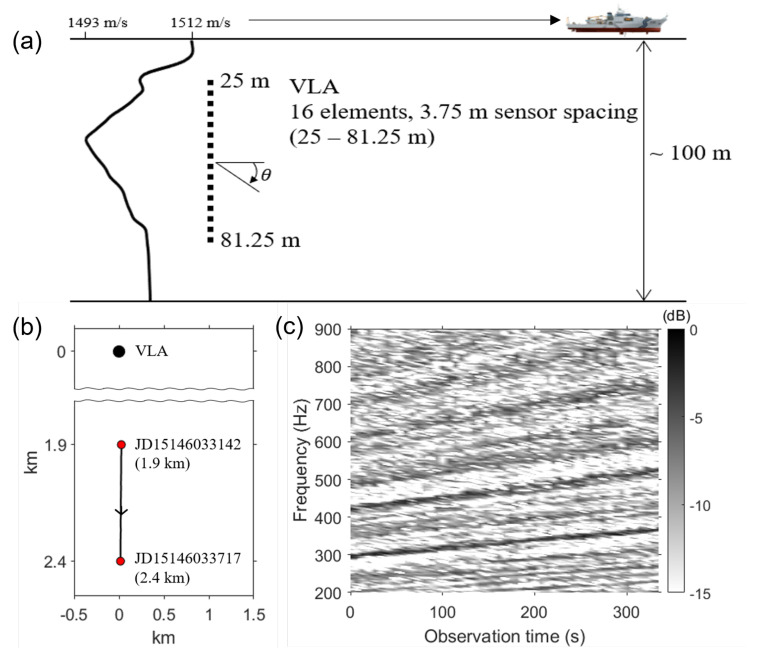
(**a**) Schematic of a source-tow run on JD 146 (26 May). A 16-element, 56.25 m long VLA was moored in apporximately 100 m deep shallow water. The SSP is an average of two CTDs collected around JD 146. (**b**) GPS ship track of the R/V *Onnuri*, radially moving away from the VLA (•) at a speed of 1.5 m/s for about 5 min. Denoted by red circles are the start (r=1.9 km, JD15146033142) and end (r=2.4 km, JD15146033717) points of the track. (**c**) Received interference patterns from the ship noise for the depth of d=51.25 m.

To investigate the improvement of Green’s functions, we analyze a dataset collected during a source-tow run on JD 146 (26 May). The schematic of the source-tow run is illustrated in Figure 3a. The bottom moored VLA consisted of 16 elements spanning a 56.25 m aperture with 3.75 m element spacing (i.e., design frequency = 200 Hz), covering approximately half the water column (from 25 to 81.25 m) in approximately 100 m deep water. The sound speed profile (SSP) displayed in Figure 3a is an average of two CTD (conductivity, temperature, and depth) casts collected around JD 146. The R/V *Onnuri* was simultaneously towing two broadband sources (3–10 kHz and 12–32 kHz) at a speed of 1.5 m/s, moving radially away from the VLA (•), as depicted in Figure 3b. The red circles denote the start (r=1.9 km, JD15146033142) and end (r=2.4 km, JD15146033717) points along the track. Our interest in this paper is the Green’s function from the R/V *Onnuri* radiating low-frequency broadband noise (<1 kHz), which did not overlap with the high-frequency waveforms broadcast by the two towed sources. Figure 3c shows the sloped striation patterns due to the constructive and destructive interference of propagating modes from the broadband ship noise (200–900 Hz) plotted in time.

## 4. Experimental Results

### 4.1. Green’s Functions from the Ship Noise

**Figure 4:**
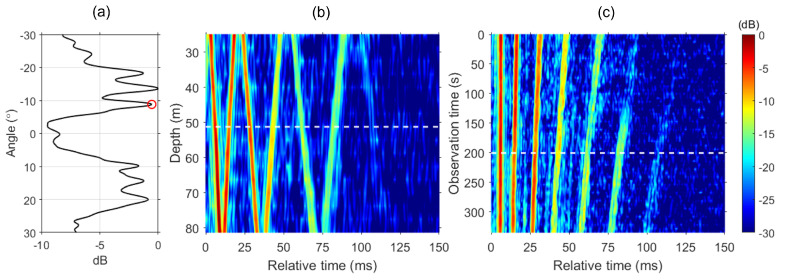
(**a**) Conventional beamformer output from a 2 s window of ship-radiated noise, averaged over the frequency band of 200–900 Hz at a reference time (200 s). Six local maxima are identified with the first arrival at $\theta =-8.8^{\circ }$ (circle). (**b**) Green’s function along the VLA depth at $r_{0}$ estimated via blind deconvolution. The six distinct wavefronts correspond to the six local maxima in (**a**). (**c**) Range-stacked Green’s functions for the depth of d=51.25 m. The horizontal line in (**c**) corresponds to the line in (**b**). The Green’s functions are aligned with respect to the first arrival with the dynamic range of 30 dB.

First, we need to estimate a Green’s function from the ship radiating broadband noise (200–900 Hz) via blind deconvolution. Displayed in Figure 4a is a conventional beamformer output applied to a 2 s ship noise at a reference observation time t0=200 s, which was averaged over the frequency band of 200–900 Hz. Six local maxima are identified with steep grazing angles ($8.8^{\circ }<\left|\theta \right|<20^{\circ }$) due to the source near the surface with a positive angle representing an up-going ray path. We then steer a beam to the first angle of arrival (red circle) and obtain the Green’s function at the reference time $g(t_{0},\tau )$ along the VLA depth, which is shown in Figure 4b. As mentioned in Section 2, τ starts from 5 ms of the earliest arrival peak, which appears at the first element (d=25 m) of the VLA, as it is the direct arrival from the ship. The six distinct wavefronts captured between 0 and 100 ms correspond to the six local maxima identified in the beamformer output in (a), while the later 7th arrival (τ>100 ms) is barely visible.

Similar to Figure 3b, we can estimate a series of Green’s functions along the radial track at approximately 5 s intervals between observation time 0 and 330 s [see Figure 3b]. Subsequently, time-stacked Green’s functions can be obtained for the middle element of the VLA whose depth is d=51.25 m, as presented in Figure 4c. The white dashed line at observation time t=200 s in Figure 4c corresponds to the time of the Green’s function in Figure 4b, while the white dashed line at the depth of d=51.25 m in Figure 4b corresponds to the depth of the Green’s function stack of Figure 4c. Note that the Green’s functions normalized at each range are aligned with respect to the first arrival. The dynamic range is 30 dB.

### 4.2. Improvement of Green’s Functions

**Figure 5:**
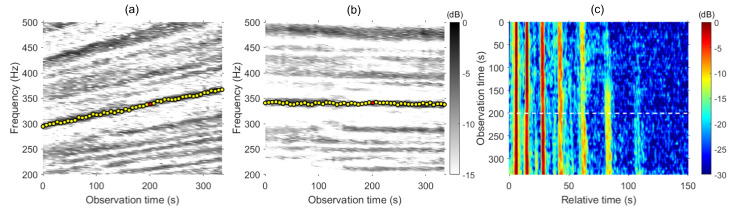
(**a**) Interference pattern from individual Green’s functions plotted at depth (d=51.25 m) versus observation time and frequency. The yellow line of circles represents the striation slope from frequency 300 to 360 Hz. The reference point (200 s, red circle) frequency is 340 Hz. (**b**) Interference pattern from individual Green’s functions extrapolated to the reference point (200 s). (**c**) Green’s functions extrapolated to the reference point (200 s, white horizontal line) at depth (d=51.25 m).

We obtain the resampling factor α=1+(Δω/ω) and the corresponding frequency shift of each point from the striation slope. The interference pattern from individual Green’s functions plotted at a depth of d=51.25 m versus observation time and frequency is illustrated in Figure 5a. The yellow line of circles represents the striation slope from 300 to 360 Hz, with the reference point (red) at a frequency of 340 Hz. The Fourier transform of individual Green’s functions extrapolated to the reference point (200 s) is shown in Figure 5b. Note that the slopes in Figure 5a have rotated to horizontal lines, indicating that the individual Green’s functions now exhibit the same intensity as the reference Green’s function. This transformation demonstrates the effectiveness of the resampling method. Figure 5c shows the Green’s functions resampled to the reference point (200 s, white horizontal line) at depth d=51.25 m. The separation of arrivals that change over time in the original [see Figure 4c] transforms to vertical straight lines through the resampling process. The results in Figure 5 clearly illustrate the success of this method, as the interference patterns show consistent intensity levels across the observation time, validating the accuracy of the frequency shifting scheme by resampling.

By eliminating the source phase from RBD with respect to the first arrival for all individual Green’s functions, the Green’s functions are perfectly aligned. This alignment allows us to ignore the time delay of individual Green’s functions, simplifying the process. Consequently, the need for prior range information is mitigated, as the alignment achieved through frequency shifts and the striation slopes ensures a coherent combination of the Green’s functions.

**Figure 6:**
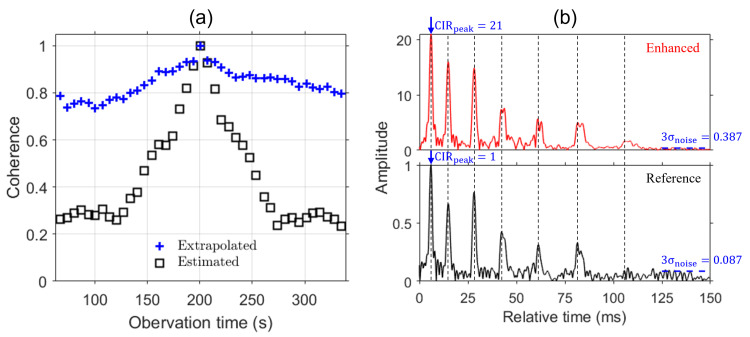
(**a**) Coherence of Green’s functions: before (squares) and after (crosses) extrapolation. The reference Green’s function is selected at $t_{0}=200$ s and d=51.25 m [horizontal line in Figure 3c]. (**b**) Normalized Green’s functions: enhanced (top) and reference (bottom). The enhancement is clearly visible in the noise floor. The enhanced Green’s function is normalized by the maximum value of the reference Green’s function.

The bottom panel of Figure 6b is the reference Green’s function at observation time t=200 s and depth d=51.25 m, corresponding to the horizontal line in Figure 5c. The similarity between the frequency-shifted neighbor Green’s functions and reference Green’s function can be measured by coherence, as depicted in Figure 6a by crosses (+). The high correlation (0.8>) for $|t-t_{0}|\leq 50$ s confirms the validity of Equation (7). On the other hand, the coherence between the original Green’s functions decreases rapidly, as indicated by squares (□) in Figure 6a [25].

After combining twenty (20) extrapolated Green’s functions of high correlation for observation time $|t-t_{0}|\leq 70$ s at approximately 5 s intervals as well as the reference, we obtain a summed Green’s function ($g_{s}$) aligned to the time of $t_{0}$ in the top panel of Figure 6b, which is in contrast to the reference Green’s function shown in the bottom. Both Green’s functions are normalized by the peak value of the reference Green’s function. Note that the arrival times are closely aligned (vertical lines): in particular, the first three ones. This indicates that the neighbor Green’s functions are properly resampled, resulting in similar arrival structures with the reference Green’s function.

To assess the enhancement of the Green’s function, we adopt the following SNR formula appropriate for impulsive signals [18,28]:

(9) $$\mathrm{SNR}=20\log\left(\frac{{\mathrm{CIR}}_{\mathrm{peak}}}{3\sigma_{\mathrm{noise}}}\right),$$

where ${\mathrm{CIR}}_{\mathrm{peak}}$ is the peak amplitude of the Green’s function. $\sigma_{\mathrm{noise}}$ is a standard deviation of the noise, which can be evaluated from the noisy segment of the Green’s function such that t>125 ms in Figure 6b. The resulting SNRs for both Green’s functions are 34 and 21 dB, respectively. The 13 dB improvement in SNR, equivalent to the array gain of 10×log(N=21), is noticeable in the lower noise floor ($3\sigma_{noise}=0.387$) in the top panel of Figure 6b compared to the noise floor ($3\sigma_{noise}=0.087$) of the reference Green’s function.

**Figure 7:**
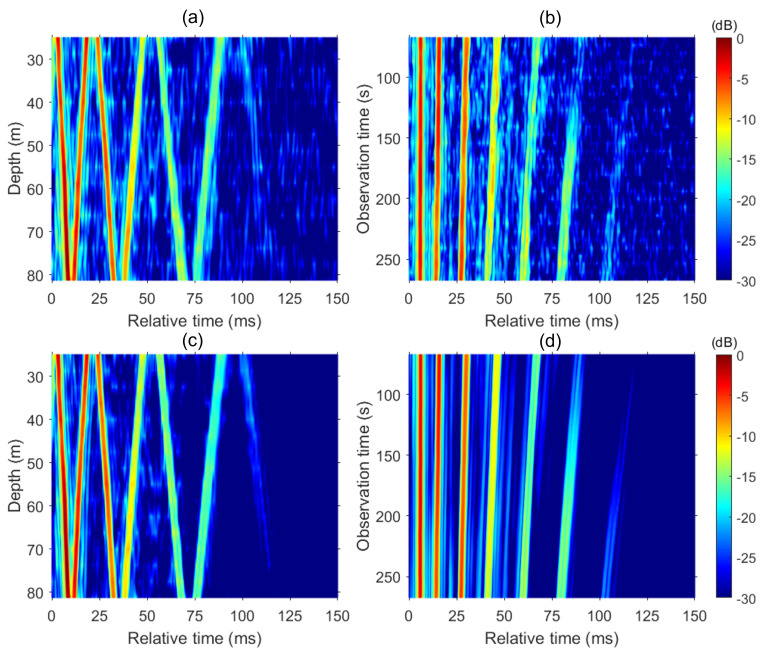
(**Top**) Same as Figure 4b,c, except that the observation time in (**b**) is limited to between 70 and 270 s for comparison with (**d**). (**Bottom**) (**c**) Enhanced Green’s function at $t_{0}=200$ s. (**d**) Enhanced range-stacked Green’s functions at d=51.25 m.

In a similar way, we can improve the Green’s function for other VLA depths and then construct an enhanced Green’s function at the reference observation time (200 s), which is presented in Figure 7c. Compared to the reference Green’s function in Figure 7a, the sidelobes and background level are much lower (i.e., blueish), and even the later 7th arrival (t>100 ms) is visible. Further, we can improve the time-stacked Green’s function at the depth d=51.25 m. For each reference point, twenty Green’s functions for $|t-t_{0}|<$ 70 s at 5 s intervals (i.e., 70 s on both sides) are extrapolated to the reference point, coherently combined, and then normalized, as displayed in Figure 7d. Note that the observation time is limited to between 70 and 260 s to keep the same number of neighboring Green’s functions. The improvement in Figure 7d is remarkable compared to Figure 7b in terms of lower sidelobes and background noise level.

**Figure 8:**
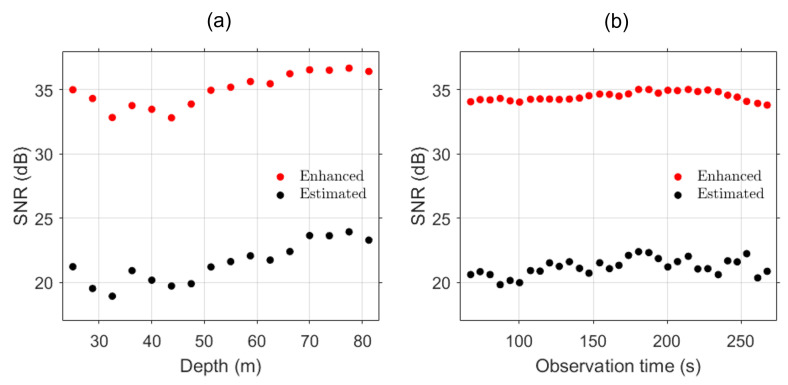
SNR of estimated (black) and enhanced (red) Green’s functions for (**a**) depth at the reference point (200 s) and (**b**) entire track at depth (d=51.25 m).

The SNR estimations at other depths of the array are depicted in Figure 8a. The red dots are the SNRs of combined Green’s functions with resampling, while the black dots indicate the SNRs of Green’s functions at the reference time. Due to the downward refracting propagation condition (see the sound speed profile in Figure 3), the SNRs are relatively higher at the deeper parts of the array. The combined Green’s functions show significant improvement compared to the reference Green’s functions. Figure 8b shows the SNR estimations along the whole track at the depth d=51.25 m. The average improvement of SNR is approximately 13 dB across depth and along the ship track. These improvements highlight the effectiveness of the extrapolation and combining method, as the enhanced Green’s functions consistently show higher SNR, validating the robustness and accuracy of the proposed approach. The increased SNR can significantly impact practical applications, ensuring clearer and more reliable acoustic signal interpretation in the specified environments.

## 5. Conclusions

This study demonstrates an improvement in the SNR of broadband Green’s functions for a moving source using the waveguide invariant theory. The proposed method effectively combines individual Green’s functions at adjacent times without prior knowledge of range or environmental parameters. Through numerical simulations and experimental data from the SAVEX15 experiment, we have shown that this approach can achieve an average of 13 dB gain in SNR.

The methodology of using RBD along with the frequency shifts obtained from striation slopes allows for a coherent combination of broadband Green’s functions, thus improving the SNR of acoustic signals in shallow-water environments. The results indicate that the proposed method can be a powerful tool for improving Green’s functions, which are critical for various underwater acoustic applications.

Future research could further investigate the application of this method in different underwater environments and with varying source movement patterns. Additionally, the integration of this technique with other acoustic signal processing methods may yield even greater improvements in source localization and acoustic sensing problems, etc.

## Figures and Tables

**Figure A1:**
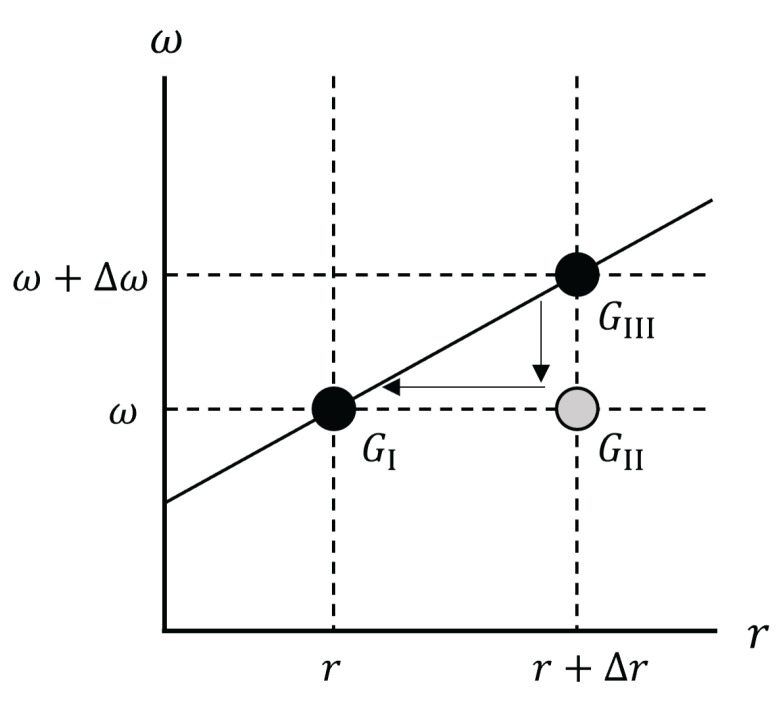
Illustration of the relationship between two Green’s functions ($G_{I}$ and $G_{\mathrm{III}}$) at adjacent range and frequency. The solid line in the frequency–range plane represents the intensity striation (β>0).

## Data Availability

Data are contained within the article.

## Appendix A. Waveguide Invariant Theory

Here, we derive Equation (6) following the conventions in Ref. [20]. The pressure field at the receiver can be written as

(A1) P(ω,r)=S(ω)G(ω,r),

where S(ω) is the source spectrum, and G(ω,r) is the Green’s function. According to normal mode theory, the Green’s function can be expressed as

(A2) $$G\left(\omega ,r\right)=\sum_{m}A_{m}\exp\left(-ik_{m}r\right),$$

where $A_{m}$ and $k_{m}$ are the *m*th mode amplitude and the horizontal wavenumber, respectively. The sound intensity can then be written as

(A3) $$I\left(\omega ,r\right)={\left|P\left(\omega ,r\right)\right|}^{2}\propto \sum_{m}{\left|A_{m}\right|}^{2}+\sum_{m\neq n}A_{m}^{*}A_{n}\exp\left(i(k_{m}-k_{n})r\right),$$

where we have removed the common factor of the source function S(ω), and $k_{m}-k_{n}$ represents the difference between pairs of horizontal wavenumbers. By ignoring the slowly varying mode amplitude term $|A_{m}{|}^{2}$, the intensity maximum is determined by the following condition:

(A4) $$\delta I\left(\omega ,r\right)\approx i\sum_{m\neq n}A_{m}^{*}A_{n}\exp\left(i(k_{m}-k_{n})\delta \right)\left(k_{m}-k_{n}\right)=0,$$

where superscript * denotes complex conjugate of $A_{m}$. The stationary phase condition can be written as

(A5) $$\delta \left[k_{m}-k_{n}\right]=\left(\frac{dk_{m}}{d\omega }-\frac{dk_{n}}{d\omega }\right)r\;d\omega +\left(k_{m}-k_{n}\right)\;dr=0.$$

This can be further expressed as

(A6) $$\begin{array}{cc} \frac{d\omega }{\omega }/\frac{dr}{r}\; & =-\left(\frac{k_{m}}{\omega }-\frac{k_{n}}{\omega }\right)/\left(\frac{dk_{m}}{d\omega }-\frac{dk_{n}}{d\omega }\right)\\ \; & =-\left(\frac{1}{v_{m}}-\frac{1}{v_{n}}\right)/\left(\frac{1}{u_{m}}-\frac{1}{u_{n}}\right)=\beta_{mn}, \end{array}$$

where $v_{m}=\omega /k_{m}$ is the phase velocity, and $u_{m}=d\omega /dk_{m}$ is the group velocity of the *m*th mode. In shallow water, $\beta_{mn}$ is approximately the same for all modes, i.e., $\beta_{mn}=\beta \approx 1$, which is known as the waveguide invariant. The waveguide invariant formulation can then be expressed as

(A7) $$\frac{\Delta \omega }{\omega }=\beta \frac{\Delta r}{r}.$$

Equation (A7), i.e., Equation (6) quantifies the slope of the interference striations in the range–frequency plane of the sound intensity fields. We use this frequency–range relationship to extrapolate the pressure field to adjacent points.

## Appendix B. Green’s Function at Adjacent Range and Frequency

This appendix describes the concept of Green’s function extrapolation following Ref. [16]. According to the waveguide invariant theory, the broadband interference structure of a sound propagated in a waveguide can be explained by the invariant β. Moving to a slightly shifted range, the same pattern of the sound field is retained but is either stretched or compressed in frequency. This property allows the Green’s function observed at one range to be extrapolated to the Green’s function at slightly shifted ranges.

Figure A1 illustrates the relationship between two Green’s functions ($G_{I}$ and $G_{\mathrm{III}}$) at adjacent range and frequency along the striation. Assuming that the same dominant modes contribute to the adjacent field along the striation, the amplitude variation can be ignored. The phase variation along the striation in Figure A1 can be written as

(A8) $$\varphi \left(r+\Delta r,\omega +\Delta \omega \right)-\varphi \left(r,\omega \right)=\bar{\gamma }\Delta \omega +\bar{k}\Delta r,$$

where $\bar{k}$ is a horizontal wavenumber, and $\bar{\gamma }$ is the group delay. The first term $\bar{\gamma }\Delta \omega$ corresponds to the phase change when $G_{\mathrm{III}}$ moves vertically down by $\Delta \omega =\beta \left(\Delta r/r\right)\omega$, which is denoted as $G_{\mathrm{II}}$ in Figure A1. The second term $\bar{k}\Delta r$ represents a phase shift over the horizontal distance Δr ($G_{\mathrm{II}}$ to $G_{I}$). From Equation (A8), the following relationship between adjacent Green’s functions is obtained as

(A9) $$\begin{array}{cc} G_{I} & =G\left(r,\omega \right)=\left[G_{\mathrm{III}}\left(r+\Delta r,\omega +\Delta \omega \right)e^{i\bar{\gamma }\Delta \omega }\right]e^{i\bar{k}\Delta r}\\ \; & \approx G_{\mathrm{II}}e^{i\omega \left(\Delta r/c\right)}. \end{array}$$

The expression in Equation (A9) can be represented in terms of the time-domain Green’s function as follows:

(A10) $$\begin{array}{cc} g(r,\omega ) & \approx g(r+\Delta r,\alpha \tau )*\delta (\tau -\Delta r/c)\\ \; & =g(r+\Delta r,\alpha (\tau -\Delta r/c)), \end{array}$$

where the resampling factor α=1+βΔr/r=1+Δω/ω is considered for the group delay ($\bar{\gamma }\Delta \omega$) and δ(τ−Δr/c) is considered for the time delay ($\bar{k}\Delta r$) in Equation (A8). In the proposed method, the unknown range *r* and r+Δr in Equation (A10) are replaced by $t_{0}$ and $t_{0}+\Delta t$, respectively. Furthermore, by selecting the same arrivals in estimating the Green’s functions via RBD, we can remove the time delay Δr/c in Equation (A10), reducing the requirement to only the frequency shift as

(A11) $$g\left(t_{0},\tau \right)\approx g\left(t_{0}+\Delta t,\alpha \tau \right).$$
